# Supplementary material for: The Role of Extracellular Vesicles (EVs) in Chronic Graft vs. Host Disease, and the Potential Function of Placental Cell-Derived EVs as a Therapeutic Tool
Source: Int J Mol Sci. 2023 May 1;24(9):8126. doi: 10.3390/ijms24098126 (PMC10179565; doi:10.3390/ijms24098126)
Supplement: Supplementary file 1 [file ijms-24-08126-s001.zip › ijms-2324015-supplementary.pdf]

International Journal of
Molecular Sciences

MDPI

Supplementary material (SM)
Supplementary Table S1: Antibodies list

Antibodies and kit for western blot	Concentration	Vender	Cat. number
Mouse anti human CD63	1:1000	abcam	ab59479
Mouse anti human CD81	1:1000	abcam	ab79559
Anti-interferon gamma (IFNγ)	1:1000	abcam	ab133566
Rabbit anti human TNF alpha	1:1000	abcam	ab215188
Rabbit anti human Placental lactogen	1:25000	abcam	ab137099
Rabbit anti human TGFβ	1:1000	abcam	ab215715
Rabbit anti human SMAD7	1:1000	abcam	ab216428
Ab5694 anti human aSMA	1:1000	abcam	Ab5694
Mouse anti human Actin	1:1000	Biomedical	691001MP
Anti-mouse	1:5000	Jackson	115-035-146
Anti-rabbit	1:5000	Jackson	111-035-144
**Antibodies for Flow Cytometry (mouse anti human)**	**Color**	**Vender**	**Cat. number**
Mouse IgG1 isotype control	FITC	BD	345815
Mouse IgG1 isotype control	PE	BD	400112
Mouse IgG1 isotype control	APC	BD	555751
Anti CD73	APC	BD	560874
Anti CD105	APC	BD	560839
Anti CD29	APC	BD	559883
Anti PDL-1	APC	BD	564715
Anti HLADR (MHC class II)	APC	BD	559868

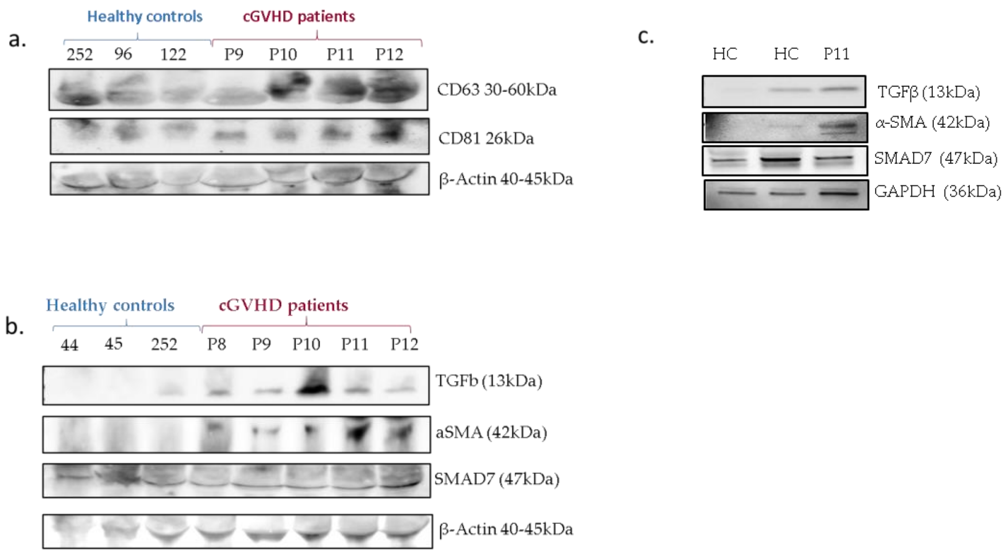

Supplementary Figure S1: Representative images of gels.

Patients and healthy controls EVs exosome markers CD63, CD81 expression compared to β-actin (a). Patients and healthy controls EVs content of fibrosis related proteins: TGF-β , SMAD7, αSMA and their control β-actin (b). Effects of EVs pellet, isolated from 150ul platelet-poor plasma (PPP) of patients with cGVHD and HC on dermal fibroblast (NHDF) cells. Fibrosis-related protein expression TGFβ , SMAD7, αSMA and their control GAPDH (c).

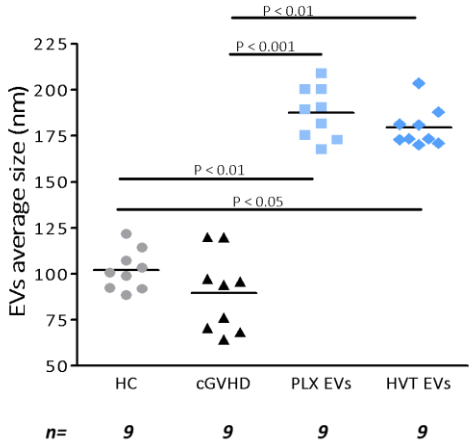

Supplementary Figure S2: EVs obtained from platelet-poor plasma (PPP) of patients with cGVHD or HC were much smaller then EVs obtained from placental cell cultures.

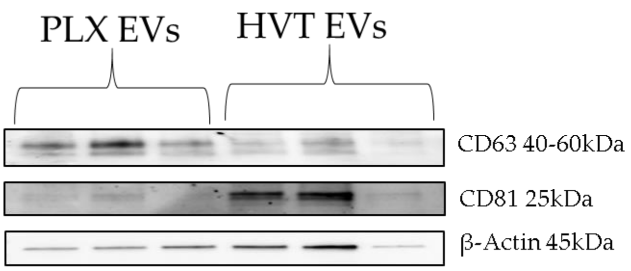

Supplementary Figure S3. Placenta EVs exosome markers expression
Representative gels of PLX and HVT EVs exosome markers CD63, CD81 and their loading control β-actin.

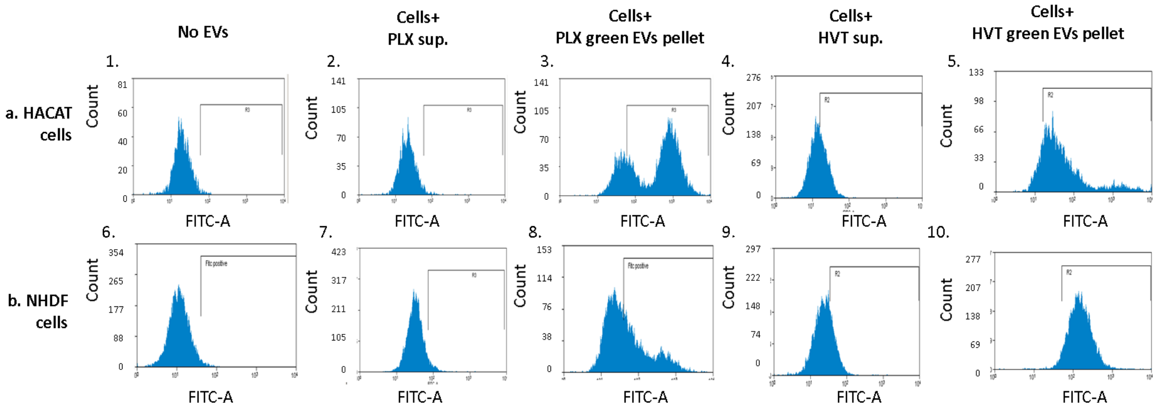

Supplementary Figure S4: Placenta EV and dermal cells interaction.
Green EV pellets isolated from Calcein-AM-labeled placental PLX and HVT cells or their supernatant fluid (SUP) were added to non-stained dermal cells: HACAT (a) and NHDF cells (b) for two hours and then washed. The cells were then analyzed with flow cytometry for the percent of FITC positive cells and compared to non-treated cells.

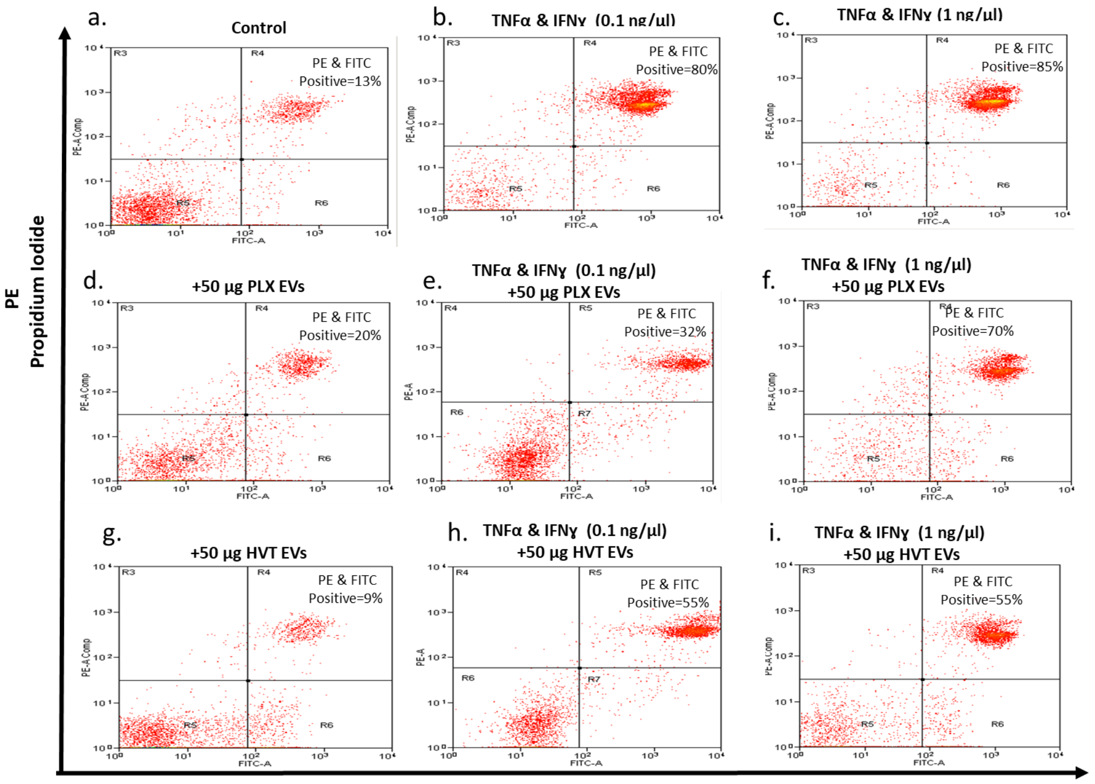

Supplementary Figure S5: The anti apoptotic effects of placental EVs on cytokines induced HACAT cells.
The effects of the cytokines on cell viability were measured after 48h of treatment and evaluated by Annexin/PI assay. Representative hystograms for : no treated cells (control) (a). Cytokines stimulated cells (TNFα and IFNɣ 0.1 and 1ng/µl) (b,c) ; cells treated with PLX Evs only (d), or combined with (TNFα and IFNɣ (0.1 and 1ng/µl) (e,f) respectivly. Cells treated with HVT Evs only (g), or combined with (TNFα and IFNɣ 0.1 and 1ng/µl) (h, i) respectivly.

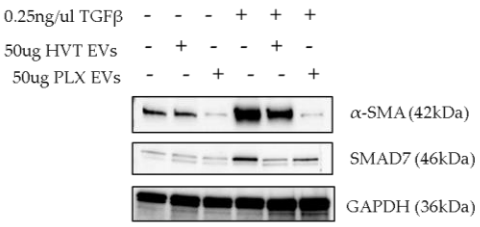

Supplementary Figure S6: Dermal fibroblast (NHDF) cells were exposed to TGFβ with or without the addition of PLX EVs or HVT EVs. The gel images of fibrotic proteins SMAD7, αSMA and their control β-actin are presented).
